# Conservation of Eelgrass (*Zostera marina*) Genetic Diversity in a Mesocosm-Based Restoration Experiment

**DOI:** 10.1371/journal.pone.0089316

**Published:** 2014-02-21

**Authors:** Brian S. Ort, C. Sarah Cohen, Katharyn E. Boyer, Laura K. Reynolds, Sheh May Tam, Sandy Wyllie-Echeverria

**Affiliations:** 1 Romberg Tiburon Center for Environmental Studies, Department of Biology, San Francisco State University, Tiburon, California, United States of America; 2 Friday Harbor Laboratories, University of Washington, Friday Harbor, Washington, United States of America; Dauphin Island Sea Lab, United States of America

## Abstract

Eelgrass (*Zostera marina*) forms the foundation of an important shallow coastal community in protected estuaries and bays. Widespread population declines have stimulated restoration efforts, but these have often overlooked the importance of maintaining the evolutionary potential of restored populations by minimizing the reduction in genetic diversity that typically accompanies restoration. In an experiment simulating a small-scale restoration, we tested the effectiveness of a buoy-deployed seeding technique to maintain genetic diversity comparable to the seed source populations. Seeds from three extant source populations in San Francisco Bay were introduced into eighteen flow-through baywater mesocosms. Following seedling establishment, we used seven polymorphic microsatellite loci to compare genetic diversity indices from 128 shoots to those found in the source populations. Importantly, allelic richness and expected heterozygosity were not significantly reduced in the mesocosms, which also preserved the strong population differentiation present among source populations. However, the inbreeding coefficient *F*_IS_ was elevated in two of the three sets of mesocosms when they were grouped according to their source population. This is probably a Wahlund effect from confining all half-siblings within each spathe to a single mesocosm, elevating *F*_IS_ when the mesocosms were considered together. The conservation of most alleles and preservation of expected heterozygosity suggests that this seeding technique is an improvement over whole-shoot transplantation in the conservation of genetic diversity in eelgrass restoration efforts.

## Introduction

Genetic diversity provides the basis for populations to respond and adapt to environmental changes and plays an important role in reducing the extinction risks associated with inbreeding [1]. The importance of maintaining genetic diversity in restoration has been recognized in many systems, such as corals [2], [3] and terrestrial plants [4], [5], and the call has been made to include genetic diversity as a measure of restoration success [6].

Seagrasses, a group of subtidal and intertidal marine angiosperms, are the foundation species for many coastal communities and provide critical habitat for commercially important fish, migrating and resident waterfowl, manatees, dugongs, and the sea turtles, as well as important ecosystem services such as stabilization of sediments, coastline protection, water purification and clarification [7], and carbon sequestration [8]. With seagrass acreage declining in recent decades, due in part to human influences [9]–[12], efforts are underway in many regions to protect existing beds and restore and expand beds into suitable, unoccupied habitat. One cosmopolitan species, Zostera marina L. (eelgrass), has been a focus of research and restoration throughout much of its range in the Northern Hemisphere [7], [13]–[18]. Positive correlations are evident between genotypic diversity in Z. marina populations and resistance to heat stress [19] and herbivory by geese [20]. However, Z. marina restoration efforts often focus on “putting plants in the ground” to maximize the area and density of coverage, while less attention is paid to simultaneously conserving and restoring genetic variation, a “keystone factor in the outcome of restoration experiments” [6]. Explicitly including the conservation of genetic diversity as one of the goals in restoration may improve the chances for long-term persistence of restored populations [21] and enhance the additional ecological benefits these plants provide [19], [20], [22], [23].

Methods that accomplish the twin goals of maximizing genetic diversity and the number of established plants are worth pursuing, especially if the program developed is ecologically viable and economically efficient. To date, restoration methods for eelgrass fall into two main categories: those that rely on clonal expansion through rhizomal growth by transplanting whole shoots, and those that involve collecting, processing and sowing seeds [14], [24]. Reliance on whole shoot transplants can result in reduced genetic diversity at the transplant site [25], which, in turn, may reduce individual fitness [26]. Using seeds for restoration takes advantage of the diversity provided by sexual reproduction [27], but introduced genotypes may differ from what once existed in a restoration site and may not benefit from localized selective advantages. Without specific knowledge about the selective properties of particular genotypes, a reasonable goal would be to use a technique that promotes genetic diversity in the restored population comparable to that of the source populations.

Large seed-broadcasting efforts require a great deal of labor and infrastructure to collect flowering shoots, float them in tanks until seeds drop, separate seeds from substrate at the bottom of the tanks, store seeds until the time to broadcast them, and coordinate the broadcasting itself. Broadcasting seeds at once also increases the potential that restoration efforts will fail due to chance events like large waves or weather conditions. Reynolds et al. [27] demonstrated successful preservation of genetic diversity in a large-scale, broadcasted-seed restoration, but for the many smaller, localized restorations, the challenges in preventing the loss of genetic diversity are especially acute. In this paper, we test the capacity of seed-based propagation to maintain genetic diversity at levels similar to those found in three source populations known to be genetically differentiated [28]. We designed a mesocosm-based study using the Buoy Deployed Seeding (BuDS) technique [24], in which spathes of *Z. marina* bearing maturing seeds are floated above the restoration area in mesh bags until, over time, matured seeds drop to the sediment surface. Compared to seed broadcasting, the method has fewer infrastructure requirements, and since seeds are released over time in a manner mimicking natural processes, it is less susceptible to failure caused by disruptive events. Therefore, BuDS should be a useful tool for small restorations if their use effectively conserves diversity.

## Materials and Methods

### Mesocosm Experimental Design

**Figure 1:**
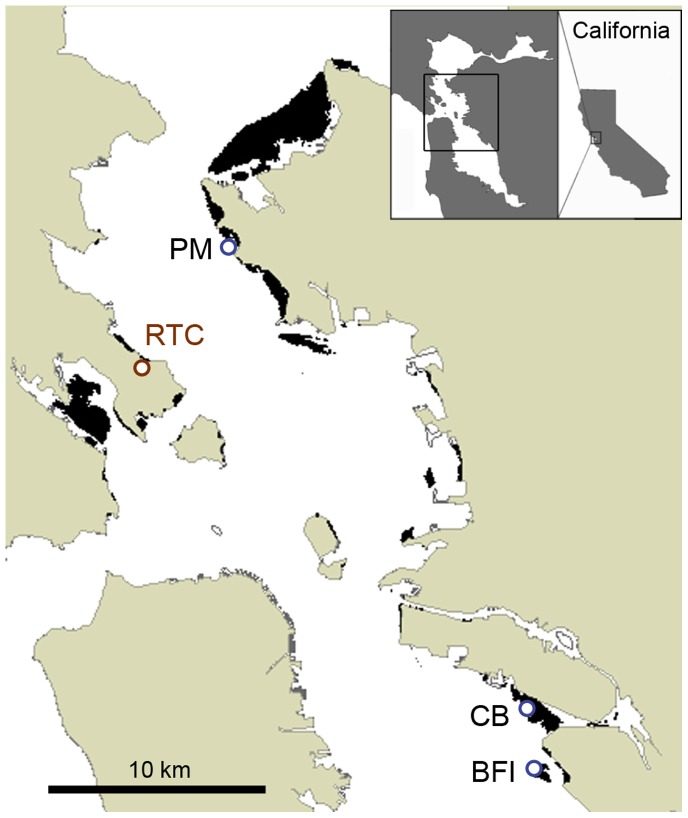
Map of the central San Francisco Bay showing locations of populations that served as the source of seed material placed in mesocosms at the Romberg Tiburon Center for Environmental Studies (RTC). Point Molate, PM; Crown Beach, CB; Bay Farm Island, BFI. Black areas indicate extent of *Z. marina* beds surveyed by side-scan sonar in 2003 [55].

This experiment was conducted in 18 mesocosms (120 cm diameter, 42 cm deep) with flow-through seawater at the Romberg Tiburon Center for Environmental Studies, central San Francisco Bay, from late July 2005 through December 2006. Salinities of the bay water near the intake averaged 26 (range 2–32) during this period, which included a very high rainfall spring. During the season of ripe seed release in late July and August 2005, we collected flowering shoots from three genetically differentiated source populations within sites known to be inhabited by *Z. marina* for several decades, and encompassing a range of environmental conditions (substrate, depth, location) and life histories (annual vs. perennial) found among the extant populations in the San Francisco Bay (Figure 1) [28], [29]. Permission to collect from the three locations was granted by the City of Richmond, California, the East Bay Regional Parks District, and the California Department of Fish and Wildlife, and the study did not involve endangered or protected species. Six mesocosms were assigned to each of the populations: one annual (Crown Beach, CB), and two perennial (Bay Farm Island, BFI, and Point Molate, PM) from different regions of the Bay, south and central, respectively (Figure S1). The bottom of each mesocosm was filled with 10 cm of clean sand from a commercial vendor. Three of the six tanks assigned to each population were inoculated with five sediment cores (5 cm diameter×5 cm deep) transported from the donor site at the same time as flowering shoot collection and spread over the sand surface. We hypothesized that introducing the native bacterial communities would allow for higher plant survival. As a control, five cores of commercial sand (as above) were added to the other three tanks. Following the Buoy-deployed Seeding (BuDS) procedures outlined by Pickerell et al. [24], a mesh pearl net containing thirty spathes with seeds in late stage 4 or 5 [30] from one of the three donor sites was suspended in the water column in each mesocosm. Pearl nets were left in the mesocosms for at least six weeks, allowing mature seeds to drop to the sediment surface. Seedlings began to appear in February 2006, followed by clonal growth. Further details of the experimental design were described in [31].

### Microsatellite Analysis

Collection of tissues occurred in June 2006, when individual clones could still be differentiated. Care was taken to ensure that sampling units were from genetically unique individuals (genets) and not clonal shoots (ramets) from the same genet. This was accomplished by sampling seedlings before rhizomes had time to expand and overlap with adjacent neighbors. Therefore, there is high confidence that the samples are unique genets within the mesocosms. We collected 100–500 mg of leaf tissue from shoots in each mesocosm and stored them in 1.5 ml microcentrifuge tubes at −80°C until DNA extraction. DNA was extracted from ∼100 mg frozen tissue from 128 samples (2–8 samples from each mesocosm, each representing a distinct genet) and scored at seven microsatellite loci, as described in [28]. Testing for null alleles was carried out by 1000 randomizations in MICROCHECKER [32]. To verify the independence of loci, we tested for linkage disequilibrium (non-independence of inheritance of alleles from two or more loci), in ARLEQUIN v3.1 [33] with a likelihood-ratio test on the genotypic data and compared the resulting statistic to a Chi-square distribution [34], using sequential Bonferroni analysis [35] on the *P*-values (α = 0.05) to reduce the likelihood of type 1 errors resulting from multiple comparisons.

### Within-population Genetic Diversity

Clonal richness and the number of multilocus genotypes were calculated in Genclone 2.0 [36]. We tested for Hardy-Weinberg equilibrium (HWE) in ARLEQUIN v3.1 [33] using the exact test of Guo and Thompson [37]. *P*-values were evaluated for significance after sequential Bonferroni adjustment [35]. We calculated allelic richness rarefied to the smallest sample size in FSTAT [38] and tested for evidence of a genetic bottleneck using the program BOTTLENECK [39] with parameters as in [28].

### Population Differentiation

Analysis of molecular variance (AMOVA) [40], implemented in ARLEQUIN v3.1 [33], was used to assess genetic differentiation among the samples, following test parameters in [28]. Fixation indices were computed by partitioning the total genetic variance into hierarchical covariance components comparing variation among individuals relative to the entire sample (*F*_IT_), inter-individual differences within a population (*F*_IS_), among populations within a group (F_SC_), and differences among groups of populations (*F*_CT_). For the purposes of these tests, each mesocosm was a “population,” and “groups” were defined as (1) all mesocosms that shared both seed source (PM, CB, or BFI) and treatment (+/− sediment inoculation), creating six groups, or (2) all mesocosms with a common seed source regardless of sediment treatment, creating three groups. Source population data for CB and BFI were obtained from Ort et al. [28], and PM data were from Tang [41].

We investigated whether individuals from the mesocosms could be genetically traced back to the correct source population using a Bayesian assignment test implemented in STRUCTURE [42]. The number of genetic clusters (populations) was set to *K* = 3, using prior knowledge of population of origin to assist clustering. Thus, the program used the three source populations to “learn” the population genotypes. We then asked the program to assign to each mesocosm sample a probability of belonging to each of the three clusters, ignoring information about the origin of the mesocosm sample. Other parameters were set at default values with GENSBACK = 1.

## Results

Seedlings had established in all 18 mesocosms by March 2006 and shoot densities were monitored monthly through December 2006, as described in [31]. Briefly, germination rates were lower for Crown Beach but clonal expansion for this donor led to marginally greater shoot densities late in the experiment. Mesocosms inoculated with sediment from the source population had higher shoot densities compared to controls for most of the experiment.

Of the 128 samples collected in June 2006, 124 samples were successfully genotyped at seven loci, and four samples yielded six loci. Null alleles and linkage disequilibrium were detected in some loci, but not in the same loci in larger samples from the source populations [28]. This result is probably due to pooling the samples across mesocosms and the generally smaller sample sizes from the mesocosms, therefore all loci were retained for analysis. There were a total of 84 multilocus genotypes (MLG) among all of the mesocosm samples. The overall genotype scoring error rate was 1.4% with a 23% resampling rate.

A summary of genetic diversity-related statistics is given in Table 1. Statistical definition of an experimental replicate depends on the question(s) tested. When testing for differences in genetic diversity among mesocosms, the mesocosm is the replicate. However, when comparing to source populations, we wanted to know if *all* genets across all six of the mesocosms for a source population captured the characteristics of the source, so the individual is the replicate unit in these tests. None of the metrics used to examine genetic diversity showed significant differences between inoculated mesocosms and controls (*N*_M_ = 3 mesocosms for each source; Table 1), except for a small but significant difference in allelic richness between the PM mesocosms (*t* = 2.45, *P*_2-tailed_ = 0.026). There was no genetic differentiation between individual mesocosms or between inoculated and control groups from the same source (Table 2), so they were pooled for comparisons among source populations. The CB mesocosms (*N*_M_ = 6) had marginally higher observed heterozygosity (*H*_O_) compared to PM (*N*_M_ = 6) (*t*-test paired by locus, *t* = 2.44, *P* = 0.05) and BFI (*N*_M_ = 6) (*t* = 2.128, *P* = 0.05). No significant difference was detected in *H*_O_ between PM and BFI (*t* = 0.905, *P* = 0.383). Expected heterozygosity (*H*_E_) was higher in the CB mesocosms than BFI (*t* = 2.86, *P* = 0.03) and trended higher compared to PM (*t* = 2.15 and, *P* = 0.07), while *H*_E_ did not differ between PM and BFI (*t* = 0.474, *P* = 0.652).

**Table 1:** Genetic diversity indices for mesocosms analyzed by source population and inoculation treatment (+/−/both treatments pooled).

Sample	Treatment	*N*_M_	*N*_I_	MLG	*R*	*AR*	*H*_O_	*H*_E_	*F*_IS_	*F*_IS_ *P*-value
PM	+	3	20	18	0.89	2.36	0.23	0.26	0.13	0.08
PM	−	3	22	15	0.67	2.22	0.14	0.21	**0.33**	0.00
PM	pooled	6	42	29	0.70	2.77	0.18	0.24	**0.22**	0.00
PM	source		47	35	0.76	2.97	0.26	0.25	−0.03	0.73
CB	+	3	24	22	0.95	2.79	0.34	0.39	**0.14**	0.01
CB	−	3	14	12	1.00	2.84	0.46	0.49	0.06	0.24
CB	pooled	6	38	34	0.97	3.26	0.36	0.40	**0.10**	0.03
CB	source		45	45	1.00	2.96	0.34	0.39	**0.13**	0.01
BFI	+	3	24	14	0.57	1.83	0.18	0.43	**0.58**	0.00
BFI	−	3	24	15	0.61	2.16	0.17	0.29	**0.44**	0.00
BFI	pooled	6	48	25	0.51	2.48	0.13	0.26	**0.50**	0.00
BFI	source		46	32	0.74	2.87	0.28	0.26	−0.05	0.82

*N*_M_, number of mesocosms; *N*_I_, number of individual ramets; MLG, number of multi-locus genotypes; *R*, clonal diversity; AR, allelic richness rarefied to smallest sample size; *H*_O_, observed heterozygosity; *H*_E_, expected heterozygosity; *F*_IS_, population inbreeding coefficient. PM source data from [41], CB and BFI source data from [28].

**Table 2:** Population pairwise *F*_ST_ for mesocosms grouped according to source population and inoculation treatment.

	PM+	PM−	CB+	CB−	BFI+	BFI−	PM source	CB source	BFI source
**PM+**	–								
**PM−**	0.01	–							
**CB+**	**0.082**	**0.125**	–						
**CB−**	**0.105**	**0.151**	−0.01	–					
**BFI+**	0.032	0.033	**0.129**	**0.154**	–				
**BFI−**	0.031	0.066*	**0.086**	**0.097**	0.011	–			
**PM source**	0.012	0.002	**0.119**	**0.14**	**0.048**	**0.062**	–		
**CB source**	**0.077**	**0.123**	0.004	−0.002	**0.105**	**0.065**	**0.114**	–	
**BFI source**	0.023	0.060*	**0.102**	**0.12**	0.012	0.005	**0.069**	**0.086**	–

Numbers in bold are significant at *P*<0.05 after sequential Bonferroni correction [35]. Numbers marked with an asterisk were significant before but not after Bonferroni correction.

Overall, allelic diversity was low among the mesocosms – only three loci, GA5, CT20 and CT3, had more than one allele present at an overall frequency greater than 0.1. At most loci, PM and BFI mesocosms had one allele at high frequency and one or more alleles at low frequency, although the low frequency alleles were not always the same for both populations. BFI mesocosms were generally the least diverse and were monomorphic at locus CT19. Allelic richness rarified to the smallest sample size revealed differences among the mesocosms only in the CB-BFI comparison (*t* = 3.02, *P* = 0.05).

Reductions in genetic diversity were small using the BuDS technique. There were no significant reductions in the number of alleles sampled, allelic richness after rarefaction, nor *H*_O_ (*t*-tests, P>0.05) between the mesocosms and their source populations (Table 1). *H*_E_ also was not significantly reduced in the mesocosms, and unexpectedly increased for CB (*t* = 4.37, *P* = 0.02). All three sets of mesocosm samples did show significantly elevated inbreeding coefficients, *F*_IS_. Among the source populations, only CB showed a significant departure from Hardy-Weinberg equilibrium (HWE) [28]. One CB mesocosm showed evidence of a genetic bottleneck (Wilcoxon test, *P*_1-tailed_ = 0.047), but this is close to what is expected by chance. No other tests in BOTTLENECK were significant.

**Figure 2:**
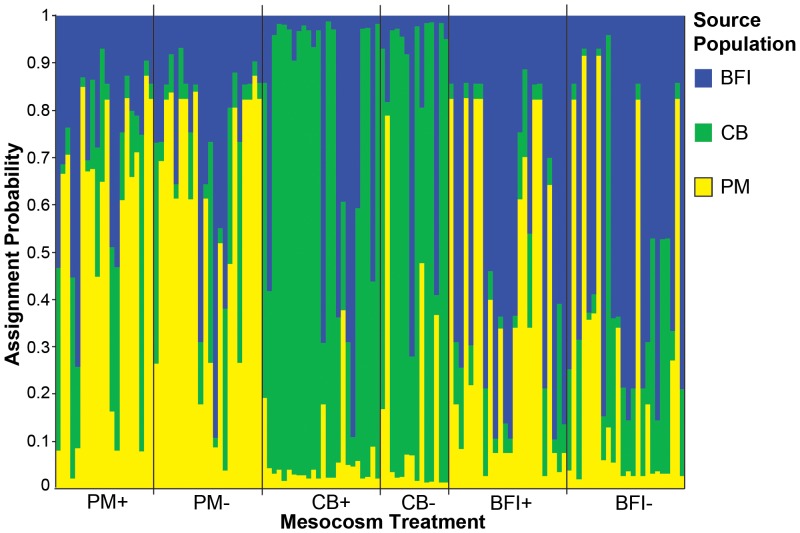
Probability of individual shoots from mesocosms being genetically assigned to one of three potential source populations, PM (sampled in 2005), CB, or BFI (both sampled in 2006), using the source populations as “learning” samples in the program STRUCTURE [42]. Each genotyped individual from the mesocosms is represented by a narrow column on the x-axis. The y-axis shows the probability of belonging to each of the three source populations.

Initial tests of genetic differentiation among individual mesocosms lacked statistical power due to small sample sizes, so we relied on indirect evidence to form conclusions regarding the genetic homogeneity among mesocosms seeded from the same donor population (see Discussion). The genetic composition of the plants in the mesocosms was similar to their respective source populations. Population differentiation, which is pronounced among the source populations [28], was maintained among the mesocosms. The proportion of the genetic variation found among mesocosms grouped by source and inoculation treatment (N_M_ = 3 mesocosms per group for 6 groups) was 6.2% (F_CT_ P<0.001), and was 8.5% when grouped only by the three sources (N_M_ = 6, F_CT_ P<0.001). In F_ST_ analyses, all three sets of mesocosms grouped by source (N_M_ = 6) were differentiated from each other but not from their respective sources (Table 3). Assignment tests carried out in STRUCTURE correctly assigned individual mesocosm shoots to their source with probability >0.5 in 84 out of 128 cases (66%), and with probability >0.7 in 58 cases (45%) (Figure 2, Table 4).

**Table 3:** Population pairwise *F*_ST_ for all mesocosms pooled according to source population regardless of inoculation treatment.

	PM pooled	CB pooled	BFI pooled	PM source	CB source	BFI source
**PM pooled**	–					
**CB pooled**	**0.114**	–				
**BFI pooled**	**0.036**	**0.113**	–			
**PM source**	0.005	**0.119**	**0.055**	–		
**CB source**	**0.109**	0.004	**0.092**	**0.114**	–	
**BFI source**	**0.041**	**0.103**	0.007	**0.069**	**0.086**	–

Numbers in bold are significant at *P*<0.05 after sequential Bonferroni correction [35].

**Table 4:** Summary of Bayesian probabilities of mesocosm samples being derived from the three source populations.

	Source population			% Correct	
Treatment	PM	CB	BFI	*Pr* >0.5	*P*r >0.7
PM pooled	30	4	8	67%	40%
CB pooled	2	27	9	71%	66%
BFI pooled	14	4	30	60%	33%

Every individual sampled from each mesocosm was estimated to have descended from each of the three possible source populations with probability *Pr*. Under the column heading for each source population is listed the number of mesocosm samples predicted to have come from that source population, based on the highest *Pr* value for that mesocosm. On the right side of the table is the percentage of mesocosm samples that were correctly assigned to their respective source populations with probability of *Pr*>0.5 or *Pr*>0.7. Probabilities were calculated using ten replicates of STRUCTURE [42] and permuted in CLUMPP [56].

## Discussion

Restoration of populations for conservation often consists of collecting propagules from an extant population and transplanting them to another location to repair damage from human activity, reestablish an extirpated population, or establish a new one in suitable habitat. The subsequent reduction in genetic diversity in the restored population can range from mild to severe, with potential effects on the long-term success of the project [1]. Using Buoy-Deployed Seeding (BuDS) [24] to deliver seeds, we successfully established new “populations” of *Z. marina* in mesocosms and maintained genetic diversity at close to the level found at *in situ* source populations, in terms of expected heterozygosity and allelic richness. In work on the Atlantic Coast, USA, Reynolds et al. [27] showed a similar ability to preserve genetic diversity in a restoration, broadcasting seeds over a larger spatial scale, while Campanella et al. [43], using a combination of seeds and transplants, found restored meadows to be genetically “healthier” than nearby natural populations. Here we demonstrate that even on a small scale, and using a different method of dispersing seeds, seed based restoration performs well at simultaneously establishing plants and conserving genetic diversity. This is an improvement over restoration programs that rely on whole shoot transplants [25]. Higher genetic diversity has been positively correlated with rates of sexual reproduction, vegetative propagation, and overall shoot density [26], and hence may contribute to the success of individual restoration efforts. While we observed reductions in heterozygosity and genotypic diversity by a factor of about 1/3 to 1/2 in the worst cases (BFI), in the best cases (CB) there was almost no reduction (Table 1). Thus, our results support the hypothesis that seed-based restoration of *Z. marina* aids in maintaining genetic diversity and may represent an improvement over exclusively transplanting whole shoots.

Shearer et al. [3] have argued that allelic richness is the most important genetic consideration in restoration, while others [1], [2] argue for including heterozygosity and genotypic richness as well. Since each of these measures has been shown to be positively correlated with ecosystem function [19], [20], [23], [26], [44], it is worthwhile to track all of them. By any of these measures, the BuDS technique performed well. Six pearl nets from each source with 30 seed-bearing spathes each was enough to capture 93% of the allelic richness detected in the PM source, 110% in the CB source, and 86% in the BFI source populations. After correcting for differences in sample sizes compared to the sources, the mesocosms captured 93% as many multi-locus genotypes from PM, 89% from CB, and 75% from BFI compared to the source populations (Table 1). Expected heterozygosity reached 96% of source levels in PM, 103% in CB, and 100% in BFI. The only exception was observed heterozygosity, which fell short of the source populations for PM (69%) and BFI (46%), while the CB mesocosms were comparable to their source (106%). In a larger restoration, the use of more BuDS units using widely spaced sampling of spathes from the source population would average out differences among individual BuDS and may further reduce the loss of diversity.

The inbreeding coefficient (*F*_IS_) was significantly elevated in the mesocosms, indicating a deficiency of heterozygotes. Heterozygosity could be reduced if a large proportion of seeds collected in the field were a result of self pollination [45]. However, parentage analysis has shown that outcrossing is a common feature of *Z. marina* populations, with evidence for multiple seed paternities within a single spathe [46], [47]. More likely, the heterozygote deficiency was due to pooling data among several mesocosms. Small sample sizes reduced the statistical power to detect differentiation among single mesocosms using pairwise estimates of *F*_ST_ (Tables 2 & 3). However, indirect evidence suggests some differentiation. Heterozygote deficiency is a hallmark of treating differentiated populations as if they are homogeneous, a phenomenon known as the Wahlund effect [48]. Reduced heterozygosity might therefore be a result of all of the seeds within each spathe being dropped in the same mesocosm. As each seed was at least a half-sibling with every other seed in the spathe, plants within the same mesocosm were more closely related to each other than to plants in other mesocosms. In this scenario, *H*_O_ would be reduced and *F*_IS_ elevated in the pooled dataset. Small, non-significant reductions in allelic richness (*AR*) are also explained in this manner. Nevertheless, pooling data across mesocosms for some analyses was required because we were interested in how an *array* of BuDS would capture the diversity of the source populations in a restoration setting and not how individual BuDS units would perform. Finally, the move from a natural environment to a laboratory setting almost certainly involved a change in selective conditions, which could contribute to a shift in allele frequencies. All of these effects could be successfully mitigated in a larger restoration attempt by using multiple BuDS from each donor population and averaging the variance associated among individual BuDS. Mitigating losses in heterozygosity is especially important in San Francisco Bay, where *Z. marina* heterozygosity levels are already relatively low compared to other populations nearby [49] and elsewhere in the Northern Hemisphere [50].

Because allelic richness diminishes more rapidly than heterozygosity after a population bottleneck event [39], it is especially encouraging to note that allelic richness was not significantly reduced in the mesocosms. In addition, patterns of population genetic structure found among the source populations [28] were replicated among the mesocosms, and an assignment test showed that most mesocosm shoots could be correctly assigned to their respective source population (Figure 2, Table 4). So it appears possible that site-specific genetic characteristics can be maintained from the source to the mesocosm populations at least for one generation. This is a salient point because it suggests that localized adaptive benefits that may exist within source populations could persist in restoration sites with similar environmental characteristics.

This study has demonstrated how experiments performed within a restoration context can provide valuable opportunities for testing hypotheses in ecology and population genetics. Gaining information about a restoration method’s effectiveness at conserving genetic diversity is a valuable tool, and similar efforts should be explicitly incorporated into programs designed to restore *Z. marina* and other aquatic plants, much as it has been for terrestrial plant restoration [4], [6], [51] and coral restoration [2], [3]. Maintaining genetic diversity in restored populations may provide a hedge against future perturbations, including the increased water temperatures predicted with climate change [52]–[54], improving the odds of long-term persistence.

## Supporting Information

Figure S1 **Design of the mesocosm experiment using Buoy Deployed Seeding (BuDS).** Flowering shoots with approximately 30 seed-bearing spathes from one of the three source populations, PM, CB, or BFI, were placed in a pearl net and floated in a mesocosm of 120 cm diameter and 42 cm depth, containing 10 cm of sand at the bottom, and with flow-through bay water. Six mesocosms were assigned to each of the source populations. Three of the six were inoculated with sediment plugs from the source location (+), and three were non-inoculated controls (−).
(DOC)
