# Supplementary material for: Conservation of Eelgrass (Zostera marina) Genetic Diversity in a Mesocosm-Based Restoration Experiment
Source: PLoS One. 2014 Feb 21;9(2):e89316. doi: 10.1371/journal.pone.0089316 (PMC3931754; doi:10.1371/journal.pone.0089316)

**Supplementary Figure S1**

Design of the mesocosm experiment using Buoy Deployed Seeding (BuDS). Flowering shoots with approximately 30 seed-bearing spathes from one of the three source populations, PM, CB, or BFI, were placed in a pearl net and floated in a mesocosm of 120 cm diameter and 42 cm depth, containing 10 cm of sand at the bottom, and with flow-through bay water. Six mesocosms were assigned to each of the source populations. Three of the six were inoculated with sediment plugs from the source location (+), and three were non-inoculated controls (-).


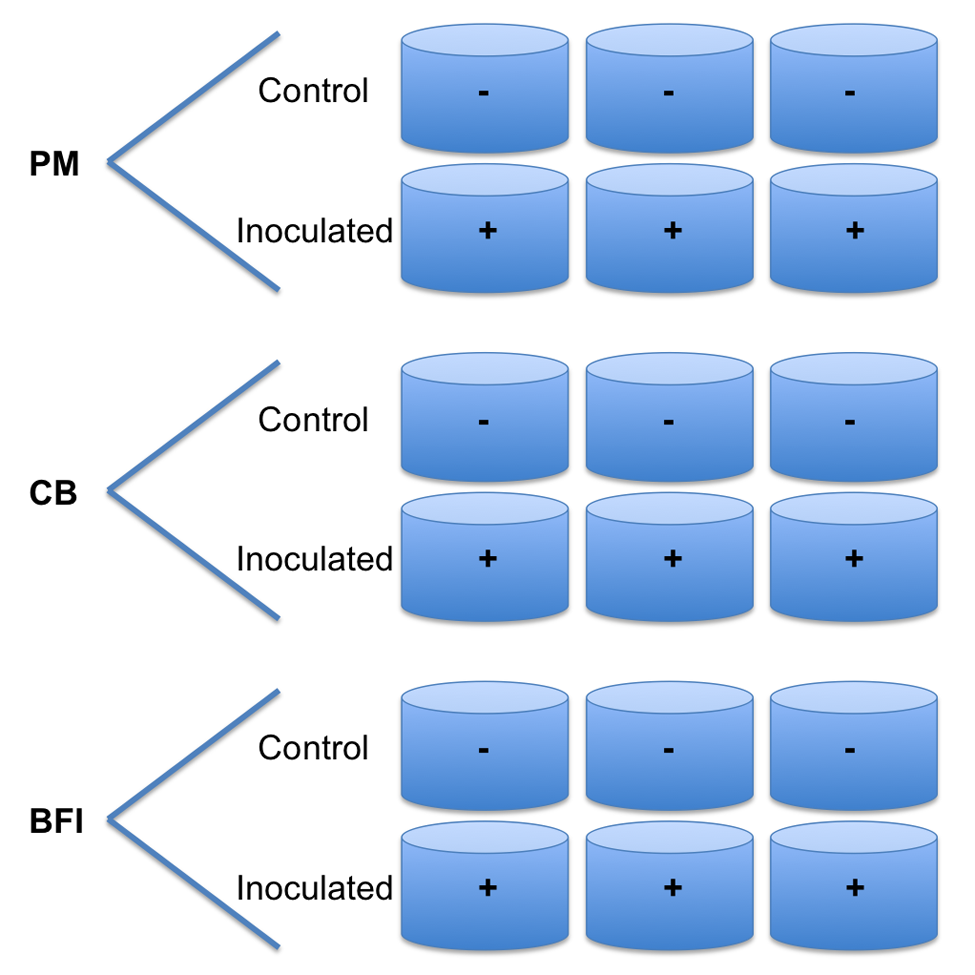

Supplement: Figure S1 — Design of the mesocosm experiment using Buoy Deployed Seeding (BuDS). Flowering shoots with approximately 30 seed-bearing spathes from one of the three source populations, PM, CB, or BFI, were placed in a pearl net and floated in a mesocosm of 120 cm diameter and 42 cm depth, containing 10 cm of sand at the bottom, and with flow-through bay water. Six mesocosms were assigned to each of the source populations. Three of the six were inoculated with sediment plugs from the source location (+), and three were non-inoculated controls (−). (DOC) [file pone.0089316.s001.doc]
